# Mistimed Feeding Disrupts Metabolic Rhythm and Increases Lipid Accumulation of Growing Rabbits in Winter

**DOI:** 10.3390/ani15050692

**Published:** 2025-02-27

**Authors:** Ke-Hao Zhang, Shuai He, Quan-Gang Wang, Jun-Jiao Li, Chun-Yan Yao, Chun-Hua Shan, Lei Zhang, Zhong-Ying Liu, Peng Liu, Ming-Yong Li, Yao Guo, Zhong-Hong Wu

**Affiliations:** 1 State Key Laboratory of Animal Nutrition, College of Animal Science and Technology, China Agricultural University, Beijing 100193, China; zhangkehao_cau@163.com (K.-H.Z.); shuaihe2021cau@126.com (S.H.); pftzdwyzc@163.com (Q.-G.W.); 17363009080@163.com (J.-J.L.); yaochunyan97@gmail.com (C.-Y.Y.); shanchh0208@163.com (C.-H.S.); zhanglei0586@foxmail.com (L.Z.); lzy228@cau.edu.cn (Z.-Y.L.); liupeng010125@163.com (P.L.); 2 National Rabbit Industry Technology System Qingdao Comprehensive Experimental Station, Qingdao 266431, China; lmy77@126.com

**Keywords:** time-restricted feeding, circadian rhythm, lipid metabolism, growing rabbits, winter

## Abstract

Maintaining the normal biological rhythms of livestock is of great significance for reflecting the environmental suitability and welfare level of animals. Mistimed feeding can interfere with the circadian rhythms of both humans and animals, resulting in disorders of lipid metabolism, obesity, and metabolic syndrome. Low-temperature environment stimulates increased appetite and decreased physical activity, resulting in higher energy intake than consumption and thus facilitating fat deposition and even obesity. In this study, growing rabbits were randomly allocated to the daytime feeding (DF) group and nighttime restricted feeding (NRF) group. Our research demonstrated that, during winter, the DF regimen disrupted the behavioral rhythms of rabbits and accelerated weight gain without changing overall feed intake. The underlying reason was that DF disturbed the lipid metabolism rhythms, promoted hepatic lipid synthesis regulated by DGAT1 and lipid synthesis of adipose tissues regulated by GPAM, thus triggering fat deposition. In contrast, the NRF regimen enhanced thermogenesis regulated by T3 and elevated body temperature and facilitated ketogenesis mediated by HMGCS2, increasing energy consumption. However, it had no significant impact on the fat content within muscle. This study offers a theoretical foundation for the refinement of feeding management and healthy raising of rabbits.

## Simple Summary

With the ceaseless advancement of intensification and automation in the rabbit industry, the implementation of precise feeding management is urgently needed. Disruptions to the feeding routine, exposure to stress, and fluctuations in environmental temperatures can upset animals’ circadian rhythms, thereby disturbing their metabolic equilibrium and impairing both health and productivity. We plan to seek ways to enhance rabbits’ welfare and production by optimizing feeding strategies. Five-week-old rabbits were assigned to the daytime feeding (DF) group and nighttime restricted feeding (NRF) group. The results proved that, during the winter, daytime feeding disrupted metabolic rhythm and increased lipid accumulation of growing rabbits in winter. In contrast, NRF significantly improved the rhythmic expression of clock genes in peripheral tissues such as the liver, muscle, and adipose tissue, ultimately reducing lipid deposition.

## 1. Introduction

With the ongoing advancements in the intensification and automation of the rabbit industry, the demand for precise feeding management has become increasingly critical. However, the common practice of daytime feeding in production does not align with the natural nocturnal feeding habit of rabbits [1]. Research shows that disruptions to the feeding schedule, along with stress and fluctuations in environmental temperature, can interfere with the circadian rhythms of animals, disrupt metabolic homeostasis, and consequently impair their health and production performance [2,3]. In addition, rabbit meat, recognized for its high protein, is favored by consumers, particularly those pursuing a healthy diet. Nonetheless, its low fat content often results in diminished flavor and tenderness.

Fat accumulation in rabbits is influenced by a multitude of factors, and studies have manifested that the characteristics of cold weather and short daylight during winter typically result in increased consumption of high-calorie foods, decreased outdoor activities, and decreased energy expenditure, subsequently leading to elevated fat deposition [4,5]. Furthermore, the circadian clock plays a crucial role in maintaining lipid metabolism homeostasis. Disruptions in circadian rhythms may alter hormone secretion patterns, including cortisol, insulin, and leptin [6,7]. Abnormal fluctuations in cortisol levels can promote fat deposition in hepatic and adipose tissues, while reduced insulin sensitivity may facilitate lipogenesis and inhibit lipolysis, culminating in increased fat accretion [8,9]. Feeding time, a crucial zeitgeber, regulates peripheral clocks and metabolic pathways in organs such as the liver, adipose tissue, and muscle [10]. Aligning the timing of nutrient intake with the body’s internal clock is instrumental in regulating lipid metabolism, maintaining glucose homeostasis, and ensuring overall energy balance [11]. Accordingly, feeding time is essential not only for the synchronization of peripheral clocks but also for the maintenance of metabolic health [12]. Time-restricted feeding (TRF), which sustains a regular feeding and fasting rhythm without reducing caloric intake, can promote daily rhythms in gene expression and prevent or reverse overweight [13]. Previous investigations conducted by our team have evidenced that, under suitable environmental conditions, night-restricted feeding (NRF) significantly improves the metabolic rhythm of the liver and enhances the production performance of growing rabbits by promoting protein synthesis and muscle growth [14]. However, previous studies were performed under optimal and stable temperature conditions, and it remains obscure whether NRF during winter can ameliorate circadian rhythms and lipid metabolism in rabbits.

According to the United Nations Food and Agriculture Organization (FAO), global rabbit meat production reached approximately 1.316 million tons in 2020. Rabbit meat serves as a substantial protein source in many regions. Moreover, it is of great significance owing to its distinct nutritional composition, featuring low cholesterol, high-quality protein, and a favorable fatty acid profile [15]. Thus, rabbits are acknowledged as economically important species in livestock farming. Rabbits, as experimental animals, share closer phylogenetic relationships with primates, and around 30% of initial vaccine development experiments involve rabbits since they are capable of mimicking human immune responses to a certain extent [16]. Understanding how different feeding patterns impinge on their biological rhythms is indispensable, as it can aid in optimizing animal health, productivity, and welfare [17,18]. Therefore, we employed growing rabbits as an experimental model to investigate the effects of daytime feeding (DF) and NRF on circadian rhythms and lipid metabolism. The aim is to explore approaches for enhancing the welfare and production performance of rabbits by optimizing their feeding management strategies and provide theoretical guidance for refining rabbit farming practices and offer valuable insights into human lipid metabolism and health.

## 2. Materials and Methods

### 2.1. Animal Feeding and Sample Collection

The study was conducted within an open-house facility equipped with rolling shutters at the Rabbit Farm of Qingdao Kangda Rabbit Industry Development Co., Ltd., located in Qingdao (35°59′ N and 119°59′ E, Qingdao, China) from 23 December 2018 to 23 February 2019. A total of 216 five-week-old weaned Hyla rabbits with the same body weight were randomly divided into two equal groups. Since sex hormones influence the fat metabolism of rabbits, females are likely to accumulate fat more easily than males [19]. Therefore, in this study, only female rabbits were used in the experiment to minimize the confounding effects associated with sex differences. The first group was the DF group (*n* = 108), in which rabbits were fed at 7:00 a.m. (ZT0, sunrise) and had ad libitum access to food throughout the day. The second group was the NRF group (*n* = 108), where rabbits were fed at 5:00 p.m. (ZT10, sunset), and their access to food was restricted from 5:00 p.m. to 7:00 a.m. The rabbits were exposed to natural light, and the daily light duration was approximately 10 h. Three rabbits were raised in one cage, and the size of the cage was approximately 60 cm × 50 cm × 37 cm. All rabbits received identical amounts of a standard diet (Table S1) and had free access to water via an automatic waterline all day; feed intake and surplus were recorded every morning. In addition, no antibiotics were used in this study. Body weight was measured weekly using a XIANGCHUAN brand electronic balance (Shanghai, China). Following the “Guide for the Care and Use of Laboratory Animals” [20], trained researchers gently removed each rabbit by placing one hand under its chest and the other supporting its hindquarters, slowly opened the cage door, weighed it on a pre-tared scale, and then returned it. At 14 weeks of age (late growth stage), 72 rabbits from each group were sacrificed by cervical dislocation at four-hour intervals over a 24-h period, starting at 11:00 a.m. Subsequently, in order to obtain samples of muscle and adipose tissues, the same experimental procedure was replicated at Qingyang (35°29′ N and 107°44′ E, Gansu, China). Serum, liver, muscle, and adipose tissue samples were collected and stored at −80 °C for subsequent analysis. Additionally, portions of liver and adipose tissue samples were fixed in 4% paraformaldehyde solution for histological examination. The experimental and animal care protocols for this study comply with the Regulations for the Administration of Laboratory Animals of the People’s Republic of China and have been approved by the Laboratory Animal Welfare and Animal Experimental Ethical Inspection of China Agricultural University (Approval Number: AW03303202-1-b-1).

### 2.2. Experimental Environment, Behavior Monitoring, and Rectal Temperature Detection

An automatic data logger (179-TH, Apresys, Duluth, GA, USA) was utilized to record the air temperature and relative humidity of the experimental rabbit house at 10-min intervals throughout the study. Rabbit behaviors were systematically monitored during the trial using an infrared camera (ds-2PT7D20IW-DE, HIKVISION, Hangzhou, China). Specifically, three rabbits from each group were selected for the observation of eating, drinking, walking, lying, and grooming behaviors from days 88 to 92, employing behavioral analysis software (The Observer XT 16.0, Noldus, Wageningen, The Netherlands). Rectal temperatures were measured hourly throughout the day using a NULAN (NULAN Animal Husbandry Technology Co., Ltd, Shijiazhuang, China) Veterinary Rectal Electronic thermometer. Before each measurement, the thermometer was cleaned with 75% alcohol and lubricated with glycerol. Measurements were taken from three rabbits every five days from each group.

### 2.3. Serum Physiological Parameters Analysis

Serum concentrations of triiodothyronine (T3) and thyroxine (T4) were determined using a radioimmunoassay method. Glucocorticoids (GC) and leptin levels in serum were measured using enzyme-linked immunosorbent assay (ELISA) kits (Thermo Multiskan Ascent, San Jose, CA, USA). Total cholesterol (TC) was quantified using an automatic biochemical analyzer detection system (Jiangsu Zecheng Biotechnology Co., Ltd., Wuxi, Jiangsu, China).

### 2.4. Lipid Analysis of the Liver

#### 2.4.1. Frozen Sections and Oil Red O Staining of the Liver

Liver tissues were cryosectioned transversely and subsequently stained for neutral lipid content using Oil Red O (ORO) as previously described. Briefly, liver samples collected at two time points (7:00 a.m. and 5:00 p.m.) were fixed in 4% paraformaldehyde, cryosectioned to 6 µm thickness, and stained for lipids with ORO (Sigma Aldrich, St. Louis, MO, USA) following the manufacturer’s protocol. The stained sections were mounted with glycerogelatin and scanned with a LEICA Pannoramic digital slide scanner (Aperio VERSA 200, Leica Microsystems, Wetzlar, Germany). The digitally captured images were subsequently processed and analyzed using ImageJ 2.x software (National Institutes of Health, Bethesda, MD, USA). Quantification of the area occupied by lipid droplets within the liver sections was performed to evaluate lipid content.

#### 2.4.2. Biochemical Detection

Hepatic triglycerides (TG), total cholesterol (TC), and β-hydroxybutyric acid (β-OHB) levels were measured using an automatic biochemical analyzer detection system (Jiangsu Zecheng Biotechnology Co., Ltd., Wuxi, Jiangsu, China) in accordance with standardized protocols. To ensure accurate comparative analysis, the obtained data were normalized to liver weight.

### 2.5. Histology and Electron Microscopy

Representative samples of perirenal adipose tissue (PRAT), white adipose tissue (WAT), and brown adipose tissue (BAT) were fixed with 4% paraformaldehyde for 24 h and subsequently stored in 70% ethanol at 4 °C. Following fixation, the tissues were embedded in paraffin, and 5-µm-thick sections were prepared using a microtome. These sections were then mounted onto slides and subjected to a baking process at 60 °C. The slides were sequentially stained with hematoxylin and eosin (Richard Allan Scientific, Kalamazoo, MI, USA) to facilitate histological examination. All stained sections were digitized using a LEICA Pannoramic digital slide scanner (Aperio VERSA 200, Leica Microsystems, Wetzlar, Germany). The digitally captured images were subsequently processed and analyzed utilizing ImageJ 2.x software.

### 2.6. RNA Extraction and Quantitative Real-Time Polymerase Chain Reaction Analyses

Gene expression was quantified using quantitative real-time polymerase chain reaction (qRT-PCR) with SYBR Green I labeling. Briefly, total RNA from liver, muscle, and adipose tissue samples was isolated using the guanidinium isothiocyanate method with Trizol Reagent (Life Technologies, Gaithersburg, MD, USA). RNA quality was assessed via agarose gel electrophoresis, and RNA quantity was determined using a biophotometer (Eppendorf, Hamburg, Germany). Following DNase treatment, first-strand complementary DNA (cDNA) synthesis was carried out using 2 μg of total RNA and random primers from the iScript cDNA Synthesis Kit (Cat# 1708890EDU, Bio-Rad, Hercules, CA, USA). Real-time PCR analysis was conducted using the Applied Biosystems 7500 Real-Time PCR System (Applied Biosystems, Foster City, CA, USA). The qRT-PCR reaction mixtures contained 12.5 μL of 2 × SYBR Green PCR Master Mix, 1.25 μL of forward and reverse primers, 1.25 μL of template cDNA, and 9.5 μL of RNase-free water (Cat# 1725201, Bio-Rad, Hercules, CA, USA). Thermal cycling conditions were as follows: an initial denaturation at 95 °C for 5 min, followed by 40 amplification cycles of 15 s at 95 °C and 20 s at 58–64 °C, with a melting curve analysis from 65 °C to 95 °C. SYBR green fluorescence was monitored at the end of each cycle to evaluate PCR product accumulation. Primer sequences are described in Table S2. Data are presented as the mean from six rabbits. The mRNA levels of target genes were normalized to glyceraldehyde-3-phosphate dehydrogenase (ΔCT) and calibrated against the control group. The relative number of target molecules was calculated using the 2^−ΔΔCT^ method, and all gene expression results are reported as the n-fold difference relative to the calibrator. The specificity of amplification products was verified.

### 2.7. Protein Preparation and Western Blot Analyses

Adipose tissue samples from rabbits were homogenized in 0.2 mL of lysis buffer [100 mM Tris (pH 7.5), 2 mM EDTA, 2 mM EGTA, 0.5 M mannitol] supplemented with 1% Triton X-100 and a protease inhibitor (Beyotime, Nanjing, China) and kept on ice during the procedure. Subsequently, the homogenate was centrifuged at 12,000× *g* for 5 min at 4 °C, and the supernatant was collected for further analysis. Protein concentration was determined using a BCA Protein Assay Reagent (Beyotime, Nanjing, China) according to the manufacturer’s protocol. Aliquots of 25 μg of protein were separated on 12% sodium dodecyl sulfate-polyacrylamide gels (Bio-Rad, Richmond, CA, USA), and the proteins were transferred onto polyvinylidene fluoride membranes (Millipore, Burlington, MA, USA) at 200 mA for 2 h in Tris-glycine buffer with 20% anhydrous ethanol at 4 °C. The membranes were blocked with Western blocking buffer (Beyotime, Nanjing, China) for 1 h at room temperature, followed by incubation with anti-PPARγ (AV32880, Sigma-Aldrich, St. Louis, MI, USA) at 4 °C overnight with gentle shaking. After thorough washing, the membranes were incubated with goat anti-rabbit secondary antibodies for 4 h at 4 °C. Membranes were also incubated with monoclonal anti-GADPH to verify equal protein loading. Western blots were developed and quantified using ImageJ 2.x software.

### 2.8. Muscle Fat Content Testing

The quadriceps muscles of rabbits at ZT4 and ZT16 were selected as test samples (*n* = 6). Small animal CT (Nemo micro CT, Pingsheng Medicals, Shanghai, China) was used to acquire the data, and parameters including tube voltage, tube current, and scanning field of view were calibrated and optimized before scanning. The sample was carefully positioned on the scanning bed, and a pre-scan was conducted to confirm the placement. Once the pre-scan was complete, an appropriate scanning region was chosen for the full scan. After scanning, the raw data were retrieved from the device workstation and processed using Avatar 1.3 software, with the window width and position adjusted to clearly delineate tissue boundaries. First, all muscle tissues were identified based on a threshold value of 460, ensuring that only muscle was selected. Next, the software isolated intramuscular fat by applying a threshold of 255. Finally, the proportion of fat in the muscle was quantitatively determined, providing an estimate of the overall fat content within the muscle tissue.

### 2.9. Statistical Analysis

To account for daily fluctuations in hormone, metabolite, and liver clock gene expression, samples collected at six distinct time points throughout the day were analyzed and treated as replicates in Student’s *t*-test using SPSS 22.0 software (SPSS Software, Chicago, IL, USA). The data for environmental temperature and humidity are presented as the mean ± standard deviation, while other data are presented as the mean ± standard error of the mean (SEM), and a *p*-value of less than 0.05 (* *p* < 0.05) was considered statistically significant. Figures were generated utilizing GraphPad Prism (version 7.00, GraphPad Software, San Diego, CA, USA). Additionally, the significance, phase, and amplitude of 24-h rhythms in some parameters were statistically evaluated using the nonparametric JTK-Cycle test implemented in R software (version 3.4.2). To visually demonstrate rhythmicity, data from the preceding day were duplicated, consistent with standard practices in circadian rhythm studies.

## 3. Results

### 3.1. Daytime Feeding Resets Behavior Rhythm and Increases Weight Gain of Rabbits in Winter

**Figure 1:**
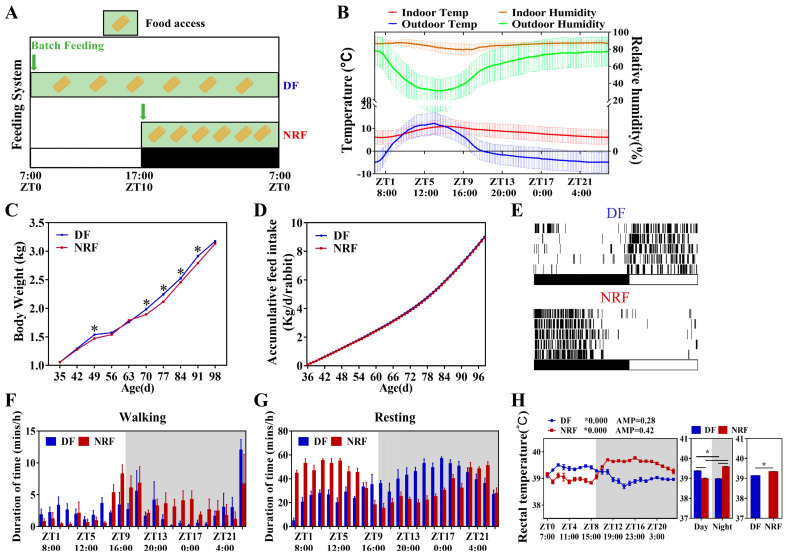
Daytime feeding disturbed the behavioral rhythm and increased weight gain in rabbits. (**A**), Design of animal feeding regimens. Rabbits subjected to DF were fed at 7:00 a.m. (ZT0) and allowed access to food all day. Rabbits under NRF had access to food from 5:00 p.m. (ZT10) to 7:00 a.m. (ZT0). ZT represents Zeitgeber Time. Green boxes denote periods of food availability. (**B**), Mean temperature and relative humidity during 24 h in the locations of the open rabbit house. (**C**), Feeding time changes the circadian rhythm of eating behavior (*n* = 3 rabbits over five days). (**D**), Accumulative feed intake (*n* = 108 per group) under DF and NRF feeding. (**E**), Average body weight of DF rabbits (*n* = 108) and NRF rabbits (*n* = 108). (**F**,**G**), Feeding time changes the circadian rhythm of walking and resting behavior. (**H**), Circadian changes in body temperature curve under DF and NRF feeding conditions (*n* = 3). The diurnal rhythms were assessed using JTK analysis, ADJ.P represents adjusted minimal *p*-values, ADJ *p* < 0.05 indicates a significant effect on circadian rhythm, AMP represents amplitude and LAG represents phase. White and gray areas correspond to objective daytime and nighttime, respectively. Statistical significance was determined using a T-test, * *p* < 0.05.

Five-week-old rabbits were fed using DF and NRF regimens (Figure 1A), with feeding times at 7:00 a.m. and 5:00 p.m., respectively. Both groups were housed in open pens under identical temperature and humidity conditions. During the experiment, the maximum ambient temperature recorded was 16.66 °C at ZT7, while the minimum temperature observed was 0.33 °C at ZT23. Relative humidity fluctuated between a peak of 94.28% at ZT3 and a nadir of 60.00% at ZT9 (Figure 1B and Figure S1). Since rabbits exhibit high sensitivity to external noise [21], feeding stimuli had an effect on rabbits’ behavior. In the NRF group, feeding behavior predominantly occurred during the nighttime. Conversely, the DF group displayed primarily daytime feeding activity, with occasional nighttime feeding, especially during the initial phase of the feeding period (Figure 1C). No significant differences were observed in daily or cumulative feed intake between the DF and NRF groups (Figure 1D). However, from day 70 to day 91, the body weight of rabbits in the DF group was significantly higher than that of the NRF group. By day 98, this difference was no longer statistically significant (Figure 1E). Analysis of body temperature revealed pronounced diurnal fluctuations in both groups. Specifically, the body temperature of the DF group was higher during the daytime and lower at nighttime, while that of the NRF group was lower during the daytime and higher at nighttime (Figure 1H), which was consistent with the observed rhythms of feeding and activity (Figure 1F,G), and the NRF group maintained significantly higher body temperatures overall. Therefore, daytime feeding during winter disrupted the behavioral rhythms of rabbits and led to increased body weight gain in rabbits.

### 3.2. Daytime Feeding Disrupts Hepatic Lipid Metabolic Rhythms and Promotes Lipid Deposition in Winter

**Figure 2:**
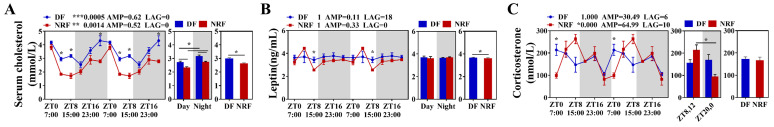
Daytime feeding altered hormonal levels in growing rabbits. (**A**–**C**) Diurnal variations in serum concentrations of cholesterol, leptin, and corticosterone in rabbits subjected to DF and NRF (*n* = 6). The diurnal rhythms were assessed using JTK analysis, ADJ *p* represents adjusted minimal *p*-values, * ADJ *p* < 0.05, indicates a significant effect on circadian rhythm, AMP represents amplitude, and LAG represents phase. White and gray areas correspond to objective daytime and nighttime, respectively. Statistical significance was determined using a T-test, * *p* < 0.05, ** ADJ *p* < 0.01, *** ADJ *p* < 0.001.

To further explore the impact of winter feeding time on lipid metabolism in rabbits, we detected the contents of cholesterol and leptin in the serum. The findings indicated that serum cholesterol and leptin levels in the DF group were significantly higher than those in the NRF group (Figure 2A,B). Moreover, the cholesterol levels in both groups exhibited diurnal fluctuations with peak levels at night and trough levels during the day, while serum leptin levels did not exhibit notable diurnal fluctuations for both groups. It is hypothesized that glucocorticoid secretion, stimulated by the low-temperature environment, may reset the peripheral circadian clock, subsequently disrupting lipid metabolism in peripheral tissues. To examine this possibility, we measured serum glucocorticoid levels and found greater fluctuations and phase shifts in corticosterone concentrations within the NRF group compared to the DF group, indicating a more pronounced diurnal rhythm (Figure 2C).

**Figure 3:**
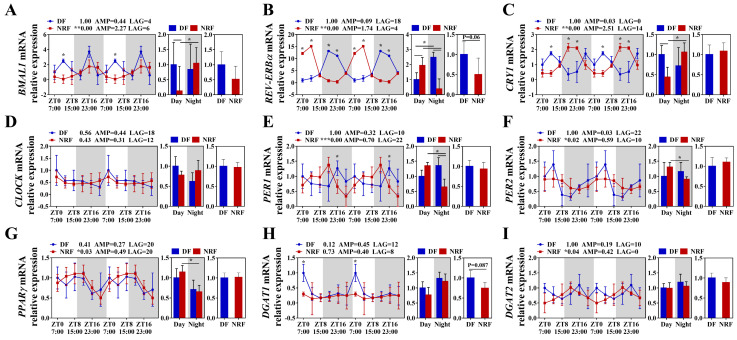
Daytime feeding disrupted circadian rhythm of clock and lipid metabolism gene in the liver. (**A**–**F**) Transcript levels of circadian genes were measured by qRT-PCR and normalized to GAPDH mRNA levels (*n* = 6). (**G**–**I**) Transcript levels of lipid metabolism genes (*n* = 6). The diurnal rhythms were assessed using JTK analysis, ADJ *p* represents adjusted minimal *p*-values, * ADJ *p* < 0.05, indicates a significant effect on circadian rhythm, AMP represents amplitude, and LAG represents phase. White and gray areas correspond to objective daytime and nighttime, respectively. Statistical significance was determined using a T-test, * *p* < 0.05, ** ADJ *p* < 0.01, *** ADJ *p* < 0.001.

**Figure 4:**
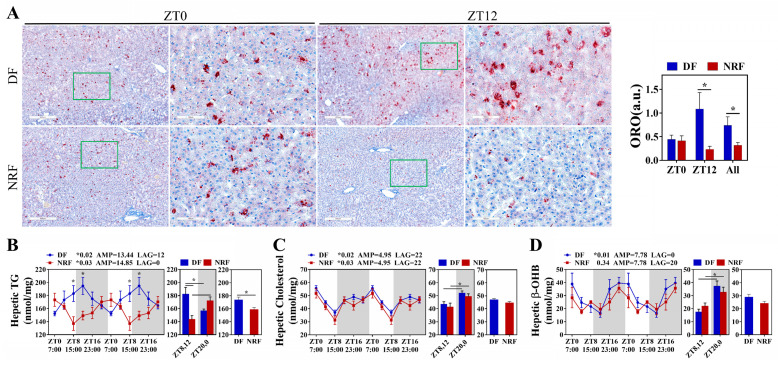
Daytime feeding increased lipid deposition in the liver. (**A**) Representative images of Oil red O-stained hepatic sections from DF and NRF rabbits (Bar = 400 µm, 100 µm). (**B**–**D**) Diurnal variations in hepatic triglyceride, cholesterol, and β-OHB concentrations in DF and NRF groups (*n* = 6). The diurnal rhythms were assessed using JTK analysis, ADJ *p* represents adjusted minimal *p*-values, * ADJ *p* < 0.05 indicates a significant effect on circadian rhythm, AMP represents amplitude and LAG represents phase. White and gray areas correspond to objective daytime and nighttime, respectively. Statistical significance was determined using a T-test, * *p* < 0.05.

Further investigation was conducted to evaluate the impact of winter feeding time on the rhythmicity of hepatic lipid metabolism. The results demonstrated that the clock genes *BMAL1*, *CLOCK*, *PER1/2*, *CRY1*, and *REV-ERBα* in the DF group did not exhibit rhythmic expression, whereas *BMAL1*, *PER1/2*, *CRY1*, and *REV-ERBα* in the NRF group displayed distinct diurnal rhythms, with significantly higher expression amplitudes (Figure 3A–F). Compared to the NRF group, the DF group exhibited an upregulation of the hepatic lipid synthesis gene *DGAT1* (Figure 3H) and a downregulation of the ketogenic rate-limiting enzyme *HMGCS2*. Moreover, DF disrupted the rhythms of lipid metabolism genes, including *PPARγ* and *HMGCS2* (Figure 3G,I). Assessment of hepatic fat content revealed that DF significantly increased hepatic fat accumulation (Figure 4A) and elevated hepatic triglyceride (TG) levels (Figure 4B). However, no significant effects were observed on hepatic cholesterol and β-hydroxybutyrate levels (Figure 4C,D). Therefore, DF was found to disrupt the rhythmicity of liver lipid metabolism, resulting in enhanced lipid deposition. Conversely, NRF may attenuate fat accumulation by promoting ketogenesis regulated by HMGCS2.

### 3.3. Daytime Feeding Disrupts the Rhythm of Thermogenesis and Promotes Lipid Deposition in Brown Adipose Tissue

**Figure 5:**
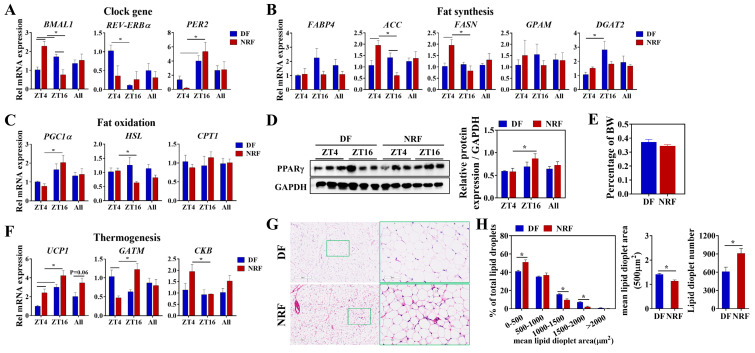
Effects of feeding time on lipid metabolism of BAT. (**A**–**C**) Transcript levels of genes associated with clock, fat synthesis, and fat oxidation in BAT from DF and NRF rabbits (*n* = 6). (**D**) Representative Western blots of PPARγ protein levels normalized to GAPDH and quantification of bands. (**E**) BAT weight was measured and normalized to body weight (BW) across the study population (*n* = 12). (**F**) Transcript levels of genes related to thermogenesis in BAT from DF and NRF rabbits (*n* = 6). (**G**) Representative images (*n* = 10) of HE-stained sections of BAT from DF and NRF rabbits (Scale bar = 400 µm or 100 µm). (**H**) Quantization of lipid droplets from (**G**) (*n* = 10). Statistical significance was determined using a T-test, * *p* < 0.05.

We investigated the effect of winter feeding time on lipid metabolic rhythms in brown adipose tissue, a specialized thermogenic tissue. Compared to the DF regimen, NRF improved the diurnal oscillations of clock genes *BMAL1* and *PER2* (Figure 5A), as well as increased the diurnal oscillations of lipid metabolism genes *PGC1*, *HSL*, *ACC*, and *FASN* (Figure 5B,C). Western blot analysis revealed that NRF elevated the oscillation of PPARγ, a critical protein involved in lipid metabolism (Figure 5D). Histological analysis demonstrated that the DF regimen significantly increased both the lipid droplet area and the number of adipocytes. Specifically, lipid droplet areas expanded to between 1000 and 2000 μm^2^ under the DF regimen (Figure 5G,H). Notably, NRF increased the diurnal oscillations of thermogenic genes *UCP1*, *GATM*, and *CKB* in BAT (Figure 5F), but there was no significant difference in the overall expression levels between the two groups. Serum analyses of thyroid hormones T3 and T4 indicated that the DF group exhibited significantly lower T3 and T4 levels at multiple time points compared to the NRF group, particularly at ZT8 and ZT16 (Figure S2), suggesting that DF reduces thyroid-induced heat production, thereby facilitating increased fat accumulation in brown adipose tissue.

### 3.4. Daytime Feeding Disrupts the Metabolic Rhythms and Facilitates Lipid Deposition in White Adipose Tissue

**Figure 6:**
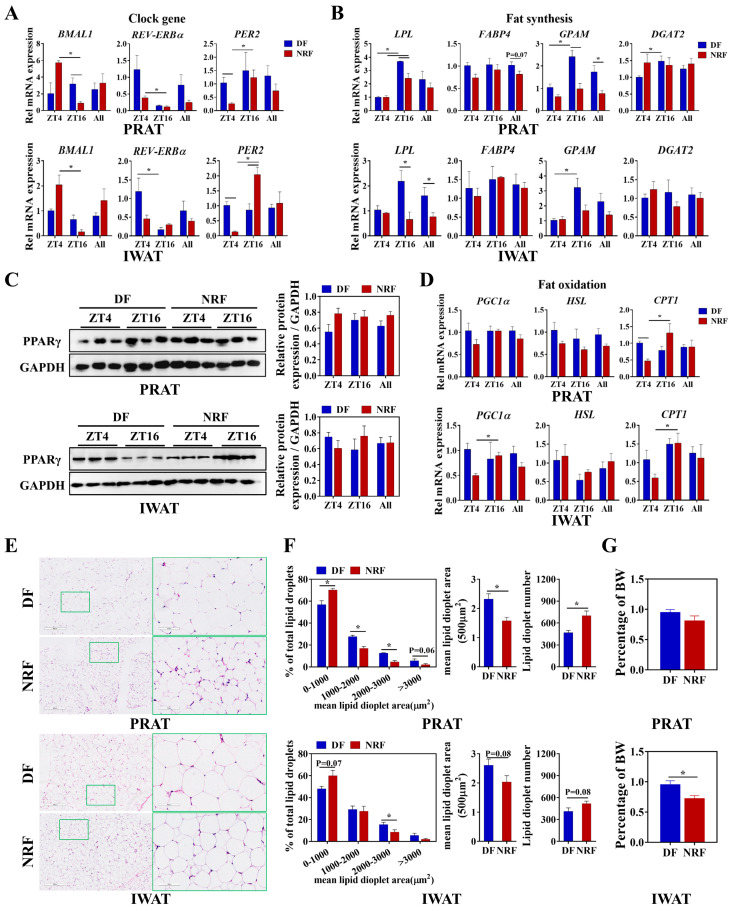
Effects of feeding time on lipid metabolism of PRAT and IWAT. (**A**,**B**,**D**) Transcript levels of genes associated with clock, fat synthesis, and fat oxidation in PRAT and IWAT from DF and NRF rabbits (*n* = 6). (**C**) Representative Western blots of PPARγ protein levels normalized to GAPDH and quantification of bands. (**E**) Representative images (*n* = 10) of HE-stained sections of PRAT and IWAT from DF and NRF rabbits (Scale bar = 400 µm and 100 µm). (**F**) Quantification of lipid droplets from (**E**) (*n* = 10). (**G**) PRAT and IWAT weight were measured and normalized to body weight (BW) across the study population (*n* = 12). Statistical significance was determined using a T-test, * *p* < 0.05.

The present study delineated the influence of winter feeding time on lipid metabolic rhythms in white adipose tissue. Compared to the DF group, NRF optimized the diurnal oscillations of clock genes *BMAL1* and *PER2* (Figure 6A), as well as the fatty acid oxidation-related gene *CPT1* (Figure 5D) in both inguinal white adipose tissue (IWAT) and perirenal adipose tissue (PRAT). Moreover, the DF upregulated the expression of the fatty acid transport gene fatty acid-binding protein 4 (*FABP4*) and the lipid synthesis gene *GPAM* in IWAT (Figure 6B), while also increasing the expression of the lipase gene *LPL* in PRAT (Figure 6B). Western blot analysis indicated no significant difference in PPARγ levels between the two groups (Figure 6C). Histological analysis revealed that DF markedly increased the lipid droplet area and the proportion of adipocytes with lipid droplet areas ranging from 2000 to 3000 μm^2^ in both IWAT and PRAT (Figure 6E,F). Additionally, the weight of IWAT was substantially increased in the DF group compared to the NRF group (Figure 6G), indicating that DF increased fatty acid uptake and synthesis, ultimately leading to increased fat deposition in white adipose tissue.

### 3.5. Daytime Feeding Disrupts Muscle Clock Gene Oscillations Without Affecting Fat Content

**Figure 7:**
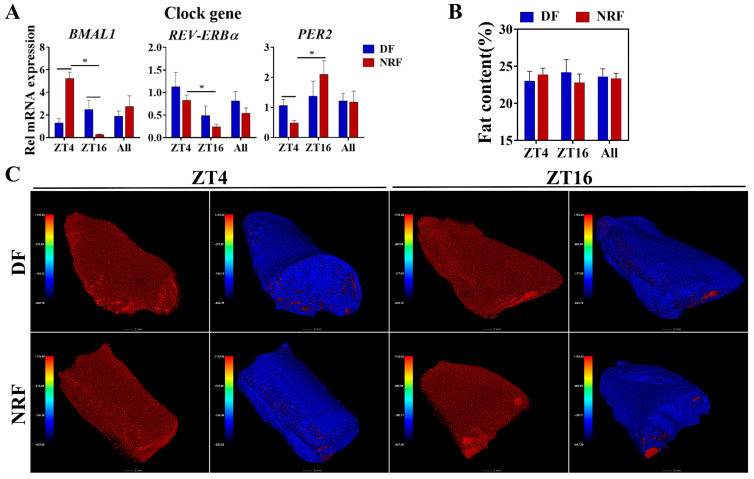
Effects of feeding time on fat content within quadriceps muscles. (**A**) Transcript levels of genes associated with clock genes in quadriceps muscles from DF and NRF rabbits (*n* = 6). (**B**) Quantification of muscle fat content in (**C**) (*n* = 12). (**C**) Representative images (*n* = 12) of CT scan images of quadriceps muscles from DF and NRF rabbits (Scale bar = 2 mm). Statistical significance was determined using a T-test, * *p* < 0.05.

To further investigate the influence of winter feeding time on muscle lipid metabolism, the expression of clock genes in muscle tissue was analyzed. The NRF group enhanced the diurnal oscillations of clock genes *BMAL1*, *REV-ERBα*, and *PER2* (Figure 7A). However, evaluation of muscle fat content indicated that there was no significant difference between the two groups (Figure 7B,C). These findings suggest that DF disrupted muscle clock gene oscillations without affecting fat content during winter.

## 4. Discussion

Feeding schedules have significant impacts on the physiological and metabolic rhythms of animals, especially under seasonal variations [22]. The timing of feeding functions as a crucial zeitgeber, influencing not only animal behaviors but also the metabolic processes across diverse tissues [23]. Previous studies have shown that feeding schedules can alter the activity of the HPA axis by regulating the release of corticotropin-releasing hormone (CRH) from the hypothalamus [24]. During periods of hunger, glucocorticoid secretion is elevated to mobilize energy reserves, thereby facilitating the organism’s adaptation to energy deficits [25]. Moreover, light exposure regulates glucocorticoid secretion by synchronizing the central biological clock in the hypothalamus, which subsequently modulates HPA axis activity [12,26]. Therefore, coordinating the feeding time with the light cycle can support glucocorticoid rhythms and thereby synchronize the biological clocks of peripheral tissues. However, exposure to cold stress during winter can trigger glucocorticoid secretion, which may disrupt the diurnal oscillation of glucocorticoids [27]. Our findings reveal that, under cold winter conditions, NRF enables rabbits to eat and walk around at night and rest during the daytime, thereby maintaining a pronounced rhythmic pattern of glucocorticoid levels. In contrast, DF causes a desynchronization between feeding rhythms and the photoperiod, resulting in altered cortisol or corticosterone levels.

The diurnal oscillation of glucocorticoids plays a pivotal role in synchronizing the circadian rhythms of peripheral tissues by binding to the glucocorticoid response element (GRE) in the promoter of Per genes [28]. This study revealed that NRF improved the rhythmic expression of clock genes in the liver, adipose tissue, and muscle, concurrently reducing lipid accumulation in both the liver and adipose tissue. Previous studies have shown that elevated REV-ERBα can directly inhibit the expression of fatty acid synthase, leading to increased expression of *CPT1B* [29]. Moreover, the overexpression of *PER2* has been shown to upregulate lipid-related genes involved in fatty acid transport and triglyceride synthesis by upregulating DGAT1 [30]. In vivo experiments revealed that *REV-ERBα*-deficient mice exhibit increased hepatic apoC-III expression, elevated plasma triglycerides (TG), and higher levels of TG-rich very low-density lipoprotein (VLDL) particles [31]. In the present study, NRF downregulated *DGAT1* expression in the liver and *GPAM* expression in inguinal and perirenal white adipose tissues, and reduced TG content in the liver and cholesterol levels in the serum. Therefore, NRF during winter enhances clock gene rhythms in peripheral tissues and attenuates lipid synthesis in both the liver and white adipose tissue.

The sympathetic activity regulated by the SCN can rhythmically regulate thermogenesis of both brown adipose tissue and muscle activity, forming the diurnal oscillation of body temperature [32,33]. According to the behavioral and body temperature results in this study, the NRF increased the body temperature of rabbits and elevated the levels of thyroid hormones T3 and T4 in the serum while also increasing activity levels. The elevated thyroid hormone levels in the serum can promote thermogenesis by upregulating the expression of *UCP1* in brown adipose tissue and also increase energy consumption in muscles [34]. Previous studies have demonstrated that the clock gene BMAL1 inhibits *UCP1* expression and attenuates BAT thermogenesis [35]. Moreover, Rev-erbα represses *UCP1* in a brown adipose cell-autonomous manner, and BAT UCP1 levels remain elevated in *REV-ERBα*-null mice even under thermoneutral conditions [36]. Genetic ablation of *REV-ERBα* also abolishes normal rhythms of body temperature and BAT activity. In this study, the NRF enhanced the nocturnal activity of rabbits and upregulated the expression of *UCP1* in brown adipose tissue, which aligns with the observed increase in nocturnal body temperature. Therefore, NRF may enhance thyroid hormone-induced thermogenesis and facilitate activity-induced energy consumption in growing rabbits during winter, thereby reducing fat deposition in brown adipose tissue.

Previous studies have demonstrated that glucocorticoids (GC) promote the differentiation of fibro-adipogenic progenitor cells (FAPs) into adipocytes through a series of signaling events [37,38]. Upon binding to their receptor GR, GC forms a hormone-receptor complex that, in conjunction with HSP90, stabilizes and maintains its activity [39]. This activation leads to the upregulation of CEBPβ, an important transcription factor that facilitates the differentiation of preadipocytes into mature adipocytes. Subsequently, CEBPβ induces the expression of *CEBPα* and *PPARγ*, both essential for adipocyte differentiation. Notably, PPARγ plays a critical role in activating adipogenic genes such as *FABP4* and *LEPTIN*, thereby initiating the adipogenesis process [40]. Additionally, glucocorticoid signaling through PPARγ and CEBPα not only promotes differentiation but also inhibits FAP proliferation by upregulating P21, which suppresses Cyclin/CDK activity [41]. However, the current study reveals that varying feeding times in winter do not alter muscle fat content. We presume that the underlying reasons are twofold. Firstly, in winter’s cold, animals prioritize thermoregulation over fat storage, channeling more energy toward heat production. Despite the GC pathway’s propensity for promoting fat formation, thermogenesis dominates, thereby halting adipocyte differentiation and maintaining muscle fat levels. Secondly, circadian clock genes might impede the GC-GR-HSP90 complex from activating C/EBPβ [42]. By governing related co-repressors, they limit *C/EBPβ* overexpression, thereby curtailing C/EBPα and PPARγ induction and preventing adipogenesis of FAPs in muscle.

Previous studies by our team have demonstrated that, under suitable environmental conditions for rabbits, the feeding time can alter the metabolic direction of nutrients. DF has been shown to promote hepatic fat synthesis, while NRF enhances production performance by stimulating protein synthesis and muscle growth in rabbits [14]. However, in this study, during winter, DF significantly elevated the production performance of rabbits before 91 days of age by increasing fat deposition. Further analysis of the metabolic direction of nutrients, under suitable environmental conditions, revealed that NRF promoted fatty acid oxidation mediated by liver CPT1B, thereby meeting the energy demands of higher activity levels. In contrast, during winter, NRF not only had to satisfy the requirements of higher activity levels but also needed to enhance UCP1-mediated brown fat thermogenesis to counteract the challenges posed by low external temperatures. Therefore, we speculate that the secretion rhythm of glucocorticoids, caused by the low-temperature environment, can alter the metabolic direction of nutrients in rabbits. However, the specific mechanism still requires further exploration.

In summary, daytime feeding during winter disrupted the lipid metabolic rhythm, increased hepatic TG content and serum cholesterol content, and increased the weight gain of rabbits by enhancing fat deposition. However, night-restricted feeding, in line with rabbits’ nocturnal habits, enhanced the diurnal oscillation of serum glucocorticoid levels. Furthermore, it also improved the rhythmic expression of clock genes in peripheral tissues such as the liver, muscle, and adipose tissue and promoted UCP1-regulated brown adipose tissue thermogenesis to resist the cold environment, which may be beneficial to the health of rabbits. However, a limitation of this study is the insufficient exploration of the specific metabolic pathways involving glucose, protein, etc. Further investigations will be conducted on the interaction mechanism between environmental temperature and feeding time, particularly in relation to glucose, lipid, and protein metabolism. By comprehensively evaluating these nutrient metabolic pathways, we aim to recommend the appropriate feeding time for rabbits, so as to improve animal welfare and enhance productivity through precise management.

## 5. Conclusions

Daytime feeding disrupted the metabolic rhythm and promoted lipid accumulation of growing rabbits in winter. However, nighttime restricted feeding ameliorated the rhythmic expression of clock genes in peripheral tissues such as the liver, muscle, and adipose tissue and reduced fat content in the liver and adipose tissue. These findings intimate a certain degree of controversy regarding the optimal feeding time for rabbits in winter. When the objective is to achieve higher production performance, it is advisable to choose daytime feeding. However, if the emphasis is on improving the welfare and health of rabbits, nighttime restricted feeding is recommended.

## Data Availability

The data presented in this study are available on request from the corresponding author. The data reflect the specific conditions of agricultural enterprises that are covered by the privacy policy.

## Supplementary Materials

The following supporting information can be downloaded at: https://www.mdpi.com/article/10.3390/ani15050692/s1, Table S1: The composition of the basic diet and nutrient content; Table S2: List of primers used for RT-PCR; Table S3: Effect of feeding time on rabbit behavior; Figure S1: The temperature and humidity in locations of the open rabbit house throughout the entire experiment; Figure S2: Feeding time alters the thyroid hormones in growing rabbits.
