# Supplementary material for: Mistimed Feeding Disrupts Metabolic Rhythm and Increases Lipid Accumulation of Growing Rabbits in Winter
Source: Animals (Basel). 2025 Feb 27;15(5):692. doi: 10.3390/ani15050692 (PMC11899554; doi:10.3390/ani15050692)
Supplement: Supplementary file 1 [file animals-15-00692-s001.zip › animals-3446193-supplementary.pdf]

Supplementary Material

Mistimed Feeding Disrupts Metabolic Rhythm and Increases Lipid Accumulation of Growing Rabbits in Winter

Ke-Hao Zhang [1], Shuai He [1], Quan-Gang Wang [1], Jun-Jiao Li [1], Chun-Yan Yao [1], Chun-Hua Shan [1], Lei Zhang [1], Zhong-Ying Liu [1], Peng Liu [1], Ming-Yong Li [2], Yao Guo [1,*] and Zhong-Hong Wu [1,*]

[1] State Key Laboratory of Animal Nutrition, College of Animal Science and Technology, China Agricultural University, Beijing 100193, China
[2] National Rabbit Industry Technology System Qingdao Comprehensive Experimental Station, Qingdao 266431, China
* Correspondence: guoyaocau@163.com (Y.G.); wuzhh@cau.edu.cn (Z.-H.W.); Tel.: +86-10-6273-2763 (Y.G. & Z.-H.W.)

Supplementary Tables and Figures

Table S1. The composition of Basic diet and nutrient content.

Raw Materials	Content	Nutritional Ingredients	Content
Alfalfa meal	31.80	Dry matter (g/kg feed)	868.00
Corn	27.60	Crude protein (g/kg DM)	202.00
Soybean meal	17.70	Neutral detergent fibre (g/kg DM)	318.00
Wheat bran	20.00	Ether extract (g/kg DM)	30.00
Premix	0.15	Digestible energy (MJ/kg DM)	11.89
Salt	0.40	Ca (g/kg DM)	10.40
Limestone	0.39	Total phosphorus (g/kg DM)	7.40
DL - Methionine	0.41	Lysine (g/kg DM)	9.80
Lysine	0.05	Methionine + cysteine (g/kg DM)	9.30
Calcium	1.30	Threonine (g/kg DM)	9.20
Threonine	0.2		
Total	100.00		

Note: Premix provided per kg of diet: 12,000 IU of vitamin A; 2500 IU of vitamin D3; 40 mg of vitamin E; 2.0 mg of vitamin K; 2.0 mg of vitamin B1; 4 mg of vitamin B2; 2.0 mg of vitamin B6; 0.01 mg of vitamin B12; 0.06 mg of biotin; 50 mg of niacin; 0.3 mg of folic acid; 10 mg of D - pantothenic acid; 1000 mg of choline; 40 mg of Zn; 10 mg of Cu; 30 mg of Mn; 50 mg of Fe; 0.5 mg of I; 0.2 mg of Se; 0.5 mg of Co.

Table S2. List of primers used for RT-PCR

Gene	Primer sequence（For）	Primer sequence（Rev）
GAPDH	TCGGAGTGAACGGATTTG	CCTGGAAGATGGTGATGG
BMAL1	GGGCTGGATGAAGACAACGA	CTAGGAGTTCCTGCGGCAAA

CLOCK	CACAGCGCAGCACTTGATAC	AAGCGAGGTTTGCTGACTGT
REV-ERBα	TGCGTACTTCCCACCATCAC	CATGGCCACCTGTAGACTCC
PER1	AGAAGGAACTCATGACGGCG	GCCTTCTTCCAGGCTCCATT
PER2	CAGCGTGAAGCAGGTGAAAG	CCACAGCAAACATATCCCCG
CRY1	CCGCCCATCACATCAGATCA	CAGCCGCCATGTACTCTTCA
PPARγ	ACGACAGACAAATCACCGTT	ATGCGGATGGCGACTTCTTT
PGC1α	CTTACTAGCACCGGCCAACA	CAACTGCGGGGTTTGTTCTG
HSL	CCAGGCTAAACTCGCATCCA	ATTTGGCTCTCTGGACTGGC
CPT1	TGGGTCTCAACACGGAACAC	GATGACTGCCTGACACTGCT
FABP4	CTAGATGGTGGTGCCCTGGT	AGTTTATCGCCCTCCCGTTT
GATM	GTACAGAGAAGCCAGGTCACA	CAGGGTTGGAAAGCACGAGA
DGAT2	AATACATCGGCTTCGCTCCC	TGTACATGGCGTGGTACAGG
DGAT1	TGTGGCCTTACTGGTGGAGT	ACCACAGGTTCACATCTCGG
UCP1	CCAAAGTCCGGCAACAGATCC	GCAACCCGCTGTAGAGTTTC
GPAM	TCAACCCCAGTATCCCGTCT	ACATTCTCGCTCACGTTGGT
CKB	CGAGGCACAGGTGGTGT	ACCATCTGCACCAGCTCC

Table S3. Effect of feeding time on rabbit behavior.

Behaviors	Items	All day		Day		Night	
		DF	NRF	DF	NRF	DF	NRF
Eating	Total time (mins/d)	197.95±9.42^{A}	231.09±10.85^{A}	196.79±8.16^{Aa}	2.32±0.15^{Bb}	5.55±1.59^{Bb}	227.44±10.83^{Aa}
	Frequency (times/d)	91.6±6.23^{A}	96.8±6.06^{A}	67±1.35^{Aa}	8±1.70^{Bb}	18.2±5.28^{Bb}	88.8±5.7^{Aa}
	Duration (mins/time)	2.37±0.17^{A}	2.41±0.14^{A}	2.76±0.19^{Aa}	0.52±0.17^{Bb}	0.38±0.06^{Bb}	2.58±0.13^{Aa}
Drinking	Total time (mins/d)	38.39±0.16^{A}	24.81±1.59^{B}	37.19±0.04^{Aa}	0.06±0.04^{Bb}	1.16±0.18^{Bb}	24.49±1.39^{Aa}
	Frequency (times/d)	68.8±6.06^{A}	43±6^{B}	62.6±6.52^{Aa}	1.2±0.58^{Bb}	4±1.22^{Bb}	41.8±5.89^{Aa}
	Duration (mins/time)	0.62±0.03^{A}	0.64±0.13^{A}	0.58±0.04^{A}	0.03±0.02^{Bb}	0.48±0.14	0.66±0.13^{a}
Walking	Total time (mins/d)	61.06±7.56^{A}	70.05±21.45^{A}	30.63±3.72^{A}	10.67±2.61^{B}	30.43±5.17	54.95±18.11
	Frequency (times/d)	160.8±24.4^{A}	113±6.74^{A}	92.2±16.64^{A}	32.2±4.23^{Bb}	69±9.23	82.5±7.31^{a}
	Duration (mins/time)	0.41±0.07^{A}	0.56±0.18^{A}	0.36±0.05	0.53±0.18	0.46±0.08	0.57±0.18
Lying	Total time (mins/d)	854.11±16.29^{A}	877.95±33.78^{A}	229.85±12.31^{Bb}	469.83±13.79^{A}	638.93±11.28^{Aa}	419.53±27.31^{B}
	Frequency (times/d)	214.2±21.47^{A}	176.8±18.31^{A}	97.2±10.4^{A}	57.8±3.2^{Bb}	117.4±11.39	119±15.37^{a}
	Duration (mins/time)	4.12±0.34^{A}	5.10±0.34^{A}	2.48±0.32^{Bb}	8.03±0.55^{Aa}	5.47±0.43^{Aa}	3.66±0.29^{Bb}

grooming	Total time (mins/d)	266.05±17.04[A]	233.88±13.06[A]	94.29±2.98[Bb]	122.26±2.62[A]	172.27±8.96[Aa]	122.71±10.31[B]
	Frequency (times/d)	282.2±31.49[A]	188±22.17[B]	132.8±22.72	68.33±1.76	139.25±6.97	118.4±18.46
	Duration **(mins/time)**	0.97±0.08[A]	1.32±0.19[A]	0.76±0.08[Bb]	1.63±0.21[A]	1.17±0.09[a]	1.15±0.21

Values are mean ± SEM, Capital letters indicate the difference between DF and NRF group at the same period, $P < 0.05$. Lowercase letters indicate the difference between day and night within the same group, $P < 0.05$.

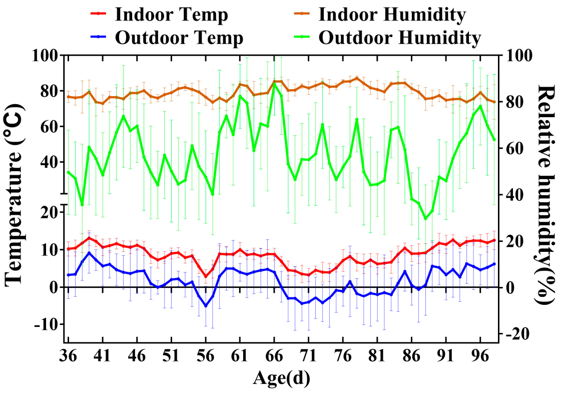

Figure S1. The temperature and humidity in locations of the open rabbit house throughout the entire experiment.

Average daily temperature and relative humidity of the open rabbit house in the whole experimental period. Values are mean ± SD.

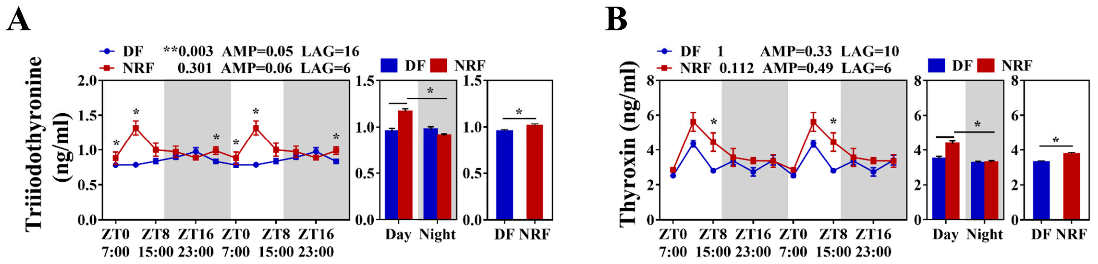

Figure S2. Feeding time alters the thyroid hormones in growing rabbits.

A and B, Diurnal variations in serum concentrations of hyroid hormones in rabbits subjected to DF and NRF (n=6). Data are presented as mean ± SEM. Statistical significance was determined using a T-test, *P<0.05.
